# Sticta Pulmonaria*Read before the Lippe Society, December 10th, 1888.

**Published:** 1889-01

**Authors:** C. Carleton Smith

**Affiliations:** Philadelphia


### STICTA PULMONARIA.*

C. Carleton Smith, M. D., Philadelphia.

As we are entering upon the season in which catarrhal diseases are very rife, I propose to call your attention to a remedy which has a most marked action upon the mucous membrane, viz., Sticta pulmonaria, which, when judiciously employed, has displayed marked curative effects in nasal and bronchial catarrhs, whether administered in the lowest, highest, or medium potencies. It is an indigenous plant, and, therefore, meets many of those cases of sudden colds ending in catarrh of the head and chest, which are so common during our changeable winter weather. And not only do we find it frequently indicated in acute attacks of the character just mentioned, but also equally efficacious in chronic nasal catarrh which has lasted many years.

All the provers felt a dull, heavy pressure in the frontal region and at root of nose, similar to that occasioned by Nux vom. This was followed by darting pains in the temporal region, burning in the eyelids with soreness of the balls in closing the lids or turning the eyes, and also marked inability to concentrate the mind. These symptoms continue to increase in severity until a cough is developed, which is very severe in its nature, hard and racking, provoked by constant tickling in the larynx, and finally extending into the chest. These symptoms, given in a general way, are the result of various provings, which, though fragmentary in their nature, have, nevertheless, enabled our school to make some important cures. But, to be more specific, and in order to get a clearer idea of the remedy, let us place the symptoms in a group as far as we are familiar with them.

Under mind we find a general confusion of ideas, the patient must talk, even though no one is listening to him. Under head we find sensation as if scalp was too small, or drawn too tight over the skull. Pains in right side of head of a darting and shooting character. Catarrhal headache, even with nausea and vomiting. Under eyes we find severe burning in the lids, with soreness of the balls, worse on closing the lids or looking from side to side. Under nose, we find that the patient wants to blow that organ constantly, but no discharge results. And the mucous membrane becomes so dry as to be quite painful, while scabs form quite rapidly, which are difficult to dislodge.

---

* Read before the Lippe Society, December 10th, 1888.

Under throat we find that the soft palate becomes so intensely dry patient cannot swallow without great pain. Now, take this group of symptoms as we have recorded them, and you cannot fail to observe a vivid picture of influenza as we so frequently meet with it in this latitude.

The cough of this drug is always dry, and invariably worse at night, preventing sleep. It is noisy and racking, accompanied with a splitting headache in frontal region. The tickling which causes the cough is so incessant that the patient soon shows sign of being completely worn out, and if not speedily controlled in some cases, becomes croupy in sound; can neither lie down* nor sleep on account of it.

There are several remedies which we may compare with Sticta, and observe at the same time the points of difference.

The stuffed feeling at the root of the nose we also find prominently under Nux vom., but this latter drug has the fluent coryza by day, and the dry coryza at night, and the three A. M. aggravation, which do not obtain under Sticta. The Sticta patient feels better in the morning and worse in the afternoon. Sensitiveness to the inspired air we also find under Rumex, Kali-b., Phos., and Dulcamara. But under Rumex the parts are so exquisitely sensitive to even the *warm air* of the room that the patient is forced to cover his head and face with the bedclothes or stuff a handkerchief in his mouth; while under Kali-b., Phos., and Dulc., the patient must needs be exposed to the cold, damp outer air in order to bring about this aggravation.

The inspired air in the case of Actea racemosa does not affect the larynx as we find under the remedies just alluded to, but the air seems to penetrate into the skull and upon the brain, causing a cold sensation.

As to the dryness of the nostrils, we must compare with Sticta Arum triphyllum, which is very important. The patient who requires Sticta, constantly blows his nose, but no discharge follows the repeated efforts. While the Arum tri. patient has a stuffed and perfectly dry nose (breathing through his mouth), yet there is a constant discharge, which excoriates the nostrils and skin of upper lip. A similar symptom is found under Nit. ac. And in differentiating further with regard to excoriating discharges, I would add just here, that Cepa excoriates the *upper lip;* Mercurius the *alæ* and *columnæ* of the nose, while Arum tri. *excoriates both nostrils and upper lip*, the *left* nostril generally the worst.

---

* Must lie down with the headache, but cannot with the cough.

There are several peculiar symptoms belonging to this drug which we will enumerate together, viz.: 1. The patient feels as if her legs were floating in the air. 2. Pain passing through from sternum to spine, with sensation as if abdomen were full of yeast, fermenting. 3. Pulsation, right side of sternum down to abdomen.

REMARKS.—A great many persons who have had sad experiences with the old school fully believe that nasal catarrh cannot be cured by any method. But, by showing our skill in the use of carefully selected homœopathic remedies, we will win many a patient over to Homœopathy. Shortly after the first fragmentary proving of Sticta was published many years ago, I obtained a vial of the tincture for the purpose of potentizing it, but, before I had the opportunity to do this, I came across a lady who was suffering most intensely with all the acute symptoms of a fully developed attack of influenza. I remarked to her that she ought to be treated for it, to which she replied that it would be of no avail, as all previous attacks had to get well of themselves, her physician being unable to afford her the slightest relief, besides informing her that catarrh could not be cured. I urged Homœopathy upon her, and, after a little persuasion, she consented to try my prescription, which consisted of a drop or two of Sticta tincture in half a glass of water, a teaspoonful once in two hours. The result was a speedy cure, to the patient's great astonishment.

A gentleman consulted me with regard to a chronic catarrh of fifteen years' standing. He explained that he was constantly blowing his nose, but no secretion took place. Besides this, dry scales frequently formed upon the mucous surface, which not only added greatly to his misery, but also prevented his smoking with any degree of comfort, on account of the obstruction to his breathing. If, said he, you can improve my condition so that I can hold a cigar in my mouth and smoke it without the necessity of removing it every moment, I will be satisfied. I put him upon Sticta, and when I was through with him he could hold a cigar between his teeth until it was reduced to the merest stump. He expressed himself as well pleased with the result. To anticipate a question which is no doubt in some of your minds, I would say, that my favorite potency in prescribing this remedy is Fincke's 6M.

December 11th, 1888.

